# Corrigendum to Cytosolic p120-catenin regulates growth of metastatic lobular carcinoma through Rock1-mediated anoikis resistance

**DOI:** 10.1172/JCI204857

**Published:** 2026-03-02

**Authors:** Ron C.J. Schackmann, Miranda van Amersfoort, Judith H.I. Haarhuis, Eva J. Vlug, Vincentius A. Halim, Jeanine M.L. Roodhart, Joost S. Vermaat, Emile E. Voest, Petra van der Groep, Paul J. van Diest, Jos Jonkers, Patrick W.B. Derksen

Original citation: *J Clin Invest*. 2011;121(8):3176–3188. https://doi.org/10.1172/JCI41695

Citation for this corrigendum: *J Clin Invest*. 2026;136(5):e204857. https://doi.org/10.1172/JCI204857

In Figure 1A of the original article, the H&E-stained images for the *Wcre;Trp53^fl/fl^* and Human ILC samples as well as the E-cadherin-stained images for the *K14cre;Cdh1^fl/fl^;Trp53^fl/fl^* and Human ILC samples were incorrect and were inadvertently duplicated from a prior publication in *Cancer* *Cell* (1). In addition, the H&E-stained image for the *K14cre;Cdh1^fl/fl^;Trp53^fl/fl^* sample and all of the p120-stained images have been replaced. The corrected figure panel, based on the original source material and data, is provided below.

The authors regret the errors.

## Figures and Tables

**Figure:**
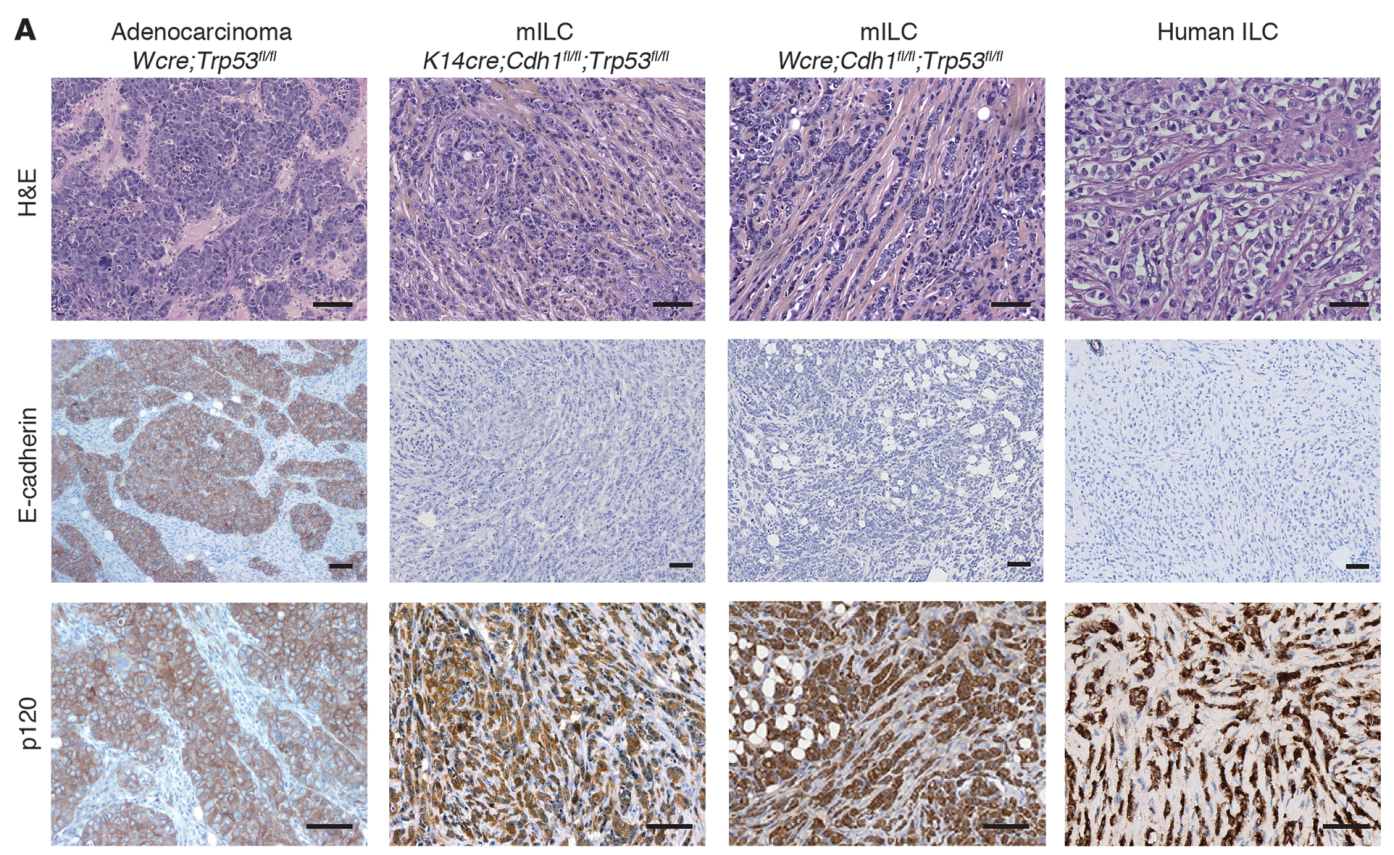


## Version 1 03/02/2026

Electronic publication
